# Long-Term Effect of Silicone Oil Tamponade for Postoperative and Posttraumatic Bacterial Endophthalmitis

**DOI:** 10.1155/2021/6658281

**Published:** 2021-02-01

**Authors:** Yong Koo Kang, Jae Pil Shin, Han Sang Park

**Affiliations:** Department of Ophthalmology, School of Medicine, Kyungpook National University, Daegu 41944, Republic of Korea

## Abstract

**Purpose:**

To compare clinical features and microbial profiles, treatment outcomes, and prognostic factors of the eyes between postoperative and posttraumatic bacterial endophthalmitis after pars plana vitrectomy (PPV) with silicone oil (SO) tamponade.

**Methods:**

Overall, 57 eyes of 57 patients who diagnosed exogenous bacterial endophthalmitis and underwent PPV with SO tamponade between 2000 and 2019 were reviewed. Causative microorganisms, culture positivity, change of mean best-corrected visual acuity (BCVA), and course of treatment were investigated between postoperative and posttraumatic groups, and relevant factors were analyzed according to the final BCVA.

**Results:**

The mean BCVA change was not significantly different between groups. The positive rate of microorganisms was significantly higher in the postoperative group. The mean time to surgery over 48 hours, initial BCVA worse than hand motion, and additional surgery after initial vitrectomy were correlated with poor final BCVA worse than 20/200. There was significantly achieved final BCVA 20/200 or better in the *Staphylococcus* and *Streptococcus* group than the *Enterococcus* and *Pseudomonas* group.

**Conclusion:**

PPV with SO tamponade may be an effective surgical treatment strategy for exogenous bacterial endophthalmitis. Final visual outcomes were not significantly different between postoperative and posttraumatic groups, and the mean time to surgery, initial visual acuity, additional surgery, and type of microorganism are significantly related to visual prognosis.

## 1. Introduction

Bacterial endophthalmitis is a serious intraocular inflammatory disease of the vitreous cavity, which is caused by the spread of infected organisms into the eye by direct inoculation, such as intraocular surgery, penetrating trauma, or hematogenous dissemination from a distant focus of infection [1]. Despite immediate treatment, it can lead to blindness and also cause irreversible damage to the photoreceptors during the infection period even if the infection is controlled. Thus, prompt diagnosis and treatment are required in maximizing outcomes [2].

Silicone oil (SO), as a substance of internal tamponade in vitreoretinal surgery, prevents the spread of infectious agents and improves the defense mechanism through a high concentration of biochemical mediators, antibodies, and inflammatory cells within the vitreous cavity [3, 4]. Moreover, SO is also used in cases of endophthalmitis with extensive retinal damage to prevent retinal detachment [4, 5]. There were a few studies on exogenous bacterial endophthalmitis treated with pars plana vitrectomy (PPV) with SO tamponade [3, 4, 6–8]. However, most of them were relatively small sample studies because of its low incidence and difficulty of long-term follow-up results.

We investigated the long-term clinical result in patients with exogenous bacterial endophthalmitis who underwent PPV with SO tamponade in the last 20 years. We compared the clinical outcomes such as causative microorganisms, culture positivity, change of mean best-corrected visual acuity (BCVA), and course of treatment in the eyes between postoperative and posttraumatic bacterial endophthalmitis and analyzed relevant factors contributing to visual prognosis.

## 2. Materials and Methods

Medical records were retrospectively reviewed after obtaining approval from the Institutional Review Board of Kyungpook National University Hospital (IRB No. 2019-10-023). The review was conducted in accordance with the tenets of the Declaration of Helsinki. Written informed consent was obtained from all participants before study enrollment.

We included patients who had exogenous bacterial endophthalmitis and underwent PPV with SO tamponade from January 2000 to May 2019 and divided them into postoperative and posttraumatic groups according to the cause of endophthalmitis. We excluded cases with the history of previous surgical intervention for endophthalmitis, treated with only intravitreal injection of antibiotics or PPV without SO tamponade, and those who had accompanied ocular pathology, such as trauma involving central cornea, which could lead to bias in visual results following surgery for endophthalmitis.

The clinical diagnosis was based on standard signs, symptoms, slit-lamp examination, and fundus examination. Smear, culture, and the sensitivity test of aqueous and vitreous humor samples subsequently confirmed the diagnosis in all patients. Clinical findings before treatment were characterized by severe hypopyon or clouding of the anterior or posterior chamber and vitreous humor, obscuring visualization of the fundus, and a diagnosis of endophthalmitis was made based on the typical clinical characteristics of patients, although confirming negative in microbial studies. Intraocular SO tamponade was performed in the eyes presenting with extensive retinal necrosis with hemorrhage and exudation that could lead to retinal detachment, intraoperative iatrogenic retinal break, or preexisting retinal detachment after clearing the anterior chamber and vitreous opacities.

A complete vitrectomy was conducted using 20-G or 23-G instruments, inducing posterior vitreous detachment, peripheral vitreous trimming with scleral depression as close to the ora serrata, and endolaser photocoagulation of any visible retinal breaks as possible. Considering the toxicity of the retina, which can be caused by antibiotic concentrations, a 500 mL balanced salt solution plus (BSS Plus®, Alcon Laboratories) dissolving vancomycin 15 mg (30 *μ*g/mL) and ceftazidime 20 mg (40 *μ*g/mL) was used during vitrectomy before fluid-air exchange [9, 10]. After fluid-air exchange, SO (Oxane®, Bausch and Lomb, USA) of 5700 centistokes (CST) was injected into the vitreous cavity. Inferior peripheral iridectomy was performed in the aphakic eyes.

All patients continued to receive empirical intravenous antibiotics and 0.5% topical moxifloxacin (Vigamox®, Alcon Laboratories, Inc., Ft Worth, TX, USA); then, antibiotics have been properly changed according to the result of the culture and sensitivity test. In addition to antibiotics, topical steroids, cycloplegics, and antiglaucoma agents were used as needed. In the eyes with reaccumulation of inflammatory cells, exudates, and hypopyon after primary surgery, additional surgical treatment was performed. The procedure of additional surgical treatment had been decided by the status of patient and surgeon's decision and performed anterior chamber irrigation of 500 mL of balanced salt solution (BSS®, Alcon Laboratories) dissolving vancomycin and ceftazidime or a half dosage intravitreal injection of vancomycin (0.5 mg/0.05 mL) and ceftazidime (1.125 mg/0.05 mL) in the SO-filled eye to avoid retinal toxicity as the limitation of the aqueous space [9–11].

Ophthalmic examinations were performed including initial best-corrected visual acuity (BCVA) using a Snellen chart, intraocular pressure (IOP), slit-lamp examination, and fundus examination. All examinations were repeated 1 week, 1 month, and 1 year postoperatively and at the final visit (later than 1 year). BCVA was converted to the logarithm of the minimum angle of resolution (logMAR) for statistical analysis, and logMAR values for low vision were defined in previous studies as follows [12]: 2.0 for counting fingers, 2.3 for hand motion, 2.6 for light perception, and 2.9 for no light perception. Additional data obtained from the medical records include sex, age, laterality of the involved eye, time from surgery to symptoms, the treatment method, and result of microbial studies.

Statistical analyses were performed using SPSS Statistics version 20 (IBM Corp., Armonk, NY, USA). Repeated measures analysis of variance corrected by the Bonferroni method was used to compare the mean BCVA according to the follow-up periods. Student's *t*-test was performed to compare the mean BCVA, and the chi-square test was performed to compare the proportion in final BCVA to groups. Clinical outcomes between groups and factors associated with final visual outcome were compared using the chi-square test, Fisher's exact test, and linear-by-linear association. For all statistical tests, a *P* value <0.05 was considered significant.

## 3. Results

A total of 85 patients who underwent PPV were initially enrolled among 183 of exogenous endophthalmitis patients, but 28 patients were excluded for failure to meet the inclusion criteria. Thus, a total of 57 patients (57 eyes) were enrolled in this study. The clinical characteristics and outcomes of all patients are summarized in Table 1.

Of 57 patients, 38 (66.7%) were male, and 19 (33.3%) were female. The mean age of all patients was 60.7 ± 18.4 years, the duration between the onset of endophthalmitis and surgery was 1.9 ± 2.5 days, and the mean follow-up period was 120.8 ± 67.4 months. 15 (26.3%) eyes were posttraumatic endophthalmitis: 2 eyes (3.5%), globe rupture; 6 eyes (10.5%), penetrating corneal injury with lens rupture; and 7 eyes (12.3%), scleral perforation with an intraocular foreign body according to the Birmingham eye trauma terminology (BETT) classification [13]. 42 eyes (73.7%) were postoperative endophthalmitis: 31 eyes (54.3%), cataract surgery; 6 eyes (10.5%), glaucoma filtering surgery; 4 eyes (7.0%), vitreoretinal surgery; and 1 eye (1.8%), intravitreal injection.

A total of 21 eyes (36.8%) underwent PPV with 20-G instruments and 36 (63.2%) with 23-G instruments. Moreover, 31 eyes (54.4%) underwent PPV including complete capsulectomy with extraction of the crystalline lens or intraocular lens (IOL). Thirteen eyes (22.8%) presented with reaccumulation of inflammatory cells, exudates, and hypopyon after primary surgery and underwent additional surgery: 8 eyes (14.0%), intravitreal injection and 5 eyes (8.8%), anterior chamber irrigation.

### 3.1. Outcomes Compared by Subgroups

Table 2 presents the clinical outcomes of the two groups. Removal of the crystalline lens or IOL and capsule in PPV was significantly more frequently performed in the posttraumatic group than in the postoperative group (*P*=0.003). Besides, microorganisms were isolated in 30 eyes (52.6%), and the positive rate was significantly higher in the postoperative group than in the posttraumatic group (*P*=0.019). The most common microorganism isolated was *Staphylococcus*, which was noted in 11 eyes (36.7%). Other cultured microorganisms included *Streptococcus* in 9 eyes (26.7%), *Enterococcus* in 8 eyes (30.0%), and Gram-negative *Pseudomonas* in 2 eyes (6.6%) according to the species and groups, as shown in Table 3.

### 3.2. Visual Outcomes

**Figure 1:**
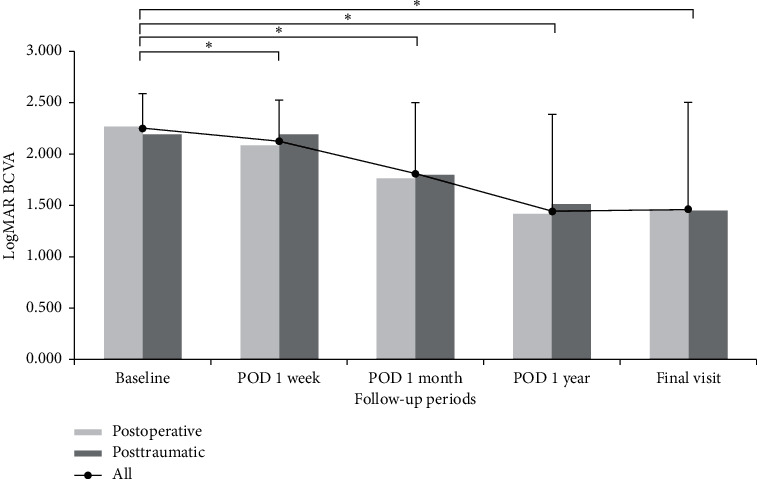
Mean logMAR (logarithm of the minimum angle of resolution) best-corrected visual acuity (BCVA) changes during follow-up. Mean BCVA was significantly improved after surgery than baseline (repeated measures analysis of variance corrected by the Bonferroni method) but not significantly different between the postoperative and posttraumatic groups during the follow-up periods (Student's *t*-test).

The change in mean BCVA was logMAR 2.283 ± 0.289 in baseline and significantly improved as 2.126 ± 0.404 after 1 week, 1.773 ± 0.743 after 1 month, 1.451 ± 0.953 after 1 year, and 1.524 ± 1.109 at the final visit (*P* < 0.001, respectively) (Figure 1). A total of 23 eyes (40.4%) achieved 20/200 or better final visual acuity, and 14 eyes (24.6%) had 20/40 or better visual acuity at the final visit. In 38 eyes with secondary SO removal surgery, the mean BCVA was significantly improved from logMAR 1.514 ± 0.602 before SO removal to 0.920 ± 0.661 at the final visit (*P* < 0.001). The mean BCVA change was not significantly different between the postoperative and posttraumatic groups during the follow-up periods (Figure 1). Moreover, 5 of 15 eyes (33.3%) in the posttraumatic group and 18 of 42 eyes (42.9%) in the postoperative group achieved 20/200 or better final visual acuity, but it was not significant (*P*=0.519).

### 3.3. Anatomical Outcomes

Only two eyes (3.5%) had progression to uncontrolled endophthalmitis after surgery and finally underwent enucleation or evisceration surgery. Moreover, 38 eyes (66.7%) underwent SO removal surgery 6.6 ± 4.5 months after primary surgery, and 24 (63.2%) of 38 aphakic eyes also underwent SO removal surgery with secondary IOL implantation. SO in 17 eyes (29.8%) was not removed because of the impossibility of obtaining a favorable visual outcome from complications. Of 17 eyes, 6 (10.5%) had redetachment with inoperable proliferative vitreoretinopathy, 6 (10.5%) had uncontrolled SO-induced secondary glaucoma, 3 (5.3%) had extensive retinal necrosis with retinal vascular obstruction, and 2 (3.5%) had severe band keratopathy from emulsified SO.

### 3.4. Relevant Factors of Final Visual Acuity

Table 4 shows the result of analyses of associations between clinical factors and final visual acuity based on 20/200. Mean time to surgery over 48 hours (*P*=0.032), initial BCVA worse than hand motion (*P*=0.007), and additional surgical treatment after initial vitrectomy (*P*=0.006) were correlated with poor final visual acuity worse than 20/200. There was no significant difference in visual outcome between culture-positive and culture-negative eyes, but final visual acuity of 20/200 or better has been significantly achieved in the *Staphylococcus* and *Streptococcus* group than in the *Enterococcus* and *Pseudomonas* group (*P*=0.014).

## 4. Discussion

Intravitreal use of antibiotics is used as the primary treatment of endophthalmitis since 1970s, and PPV is recently considered the standard treatment of endophthalmitis [10]. Nevertheless, the usage of intravitreal antibiotics is also necessary for surgical treatment of endophthalmitis. Although the positive clinical outcome could be attributed to the effect of antibiotics, we suggested that SO tamponade could be also attributed in additional roles for treatment of endophthalmitis.

The potential of SO in the treatment of endophthalmitis has been reported. Özdamar et al. demonstrated in vitro that SO decreases the proliferation of bacteria responsible for endophthalmitis [14]. It could be explained by the action of SO, which is promoting toxicity to the bacteria. It also could be explained that highly hydrophobic SO with a high interfacial tension presents a low permeability to the passage of inflammatory cells and infectious agents [15]. Siqueira et al. performed PPV with SO tamponade in uncontrolled endophthalmitis despite intravitreal antibiotic injection treatment and presented favorable outcomes and suggested that SO limits the space for the movement of infectious agents and comes in contact with the ciliary body and retina vessels. Moreover, it can enhance the effectiveness of the defense mechanisms by high concentrations of biochemical mediators, antibodies, and inflammatory cells within the aqueous compartment [4]. Especially, the prone position after SO tamponade concentrates the vitreous fluid, including infectious agents and inflammatory mediators, to the anterior part of the vitreous cavity or anterior chamber. On the contrary, SO prevents the movement and proliferation of infectious agents and concentrates the defense mechanisms against infectious agents, as it can protect the macula, which is vulnerable to antibiotic toxicity [3, 4, 7].

In the literature, retinal detachment is a frequent complication of endophthalmitis. There were various reports about the incidence rate of 4.6%–16% in concurrent or delayed-onset retinal detachment with endophthalmitis [16–18]. Other studies also reported that concurrent endophthalmitis and retinal detachment have poor visual and anatomical prognoses, especially when retinal detachment is an intraoperative complication [19–21]. In this study, 10.5% of patients developed redetachment with proliferative vitreoretinopathy, which was similar to the previous reports. SO tamponade that prevents retinal detachment may be controversial, especially inferior retinal detachment. However, Aras et al. [6] reported in a small case series of retinal detachment associated with endophthalmitis that one patient had a final visual outcome of 20/40 after removal of SO. In addition, Dave et al. [18] analyzed the prognosis after PPV with SO tamponade in patients with endophthalmitis and retinal detachment, and 93% of anatomical success and 37.6% of visual outcome over 20/400 were confirmed. According to these results, SO tamponade may be a useful option in the management of concurrent or delayed-onset retinal detachment in endophthalmitis.

According to the endophthalmitis vitrectomy study (EVS), 82% of patients achieved 20/200 or better final visual acuity and 53% achieved 20/40 or better following PPV [16]. This study showed improved visual acuity postoperatively, but the proportion of patients with visual acuity >20/200 or 20/40 was lower than those in the EVS results. However, it depended on regional characteristics and the proportion of patients with final visual acuity of 20/40 or better varied from 7.0% to 64.6% [22–25]. It was known that posttraumatic endophthalmitis had a poor visual outcome [26–28]. However, there was no significant difference in BCVA change and the final visual outcome between postoperative and posttraumatic groups in this study. We already excluded cases that may affect the analysis of visual acuity and analyzed the effect of endophthalmitis itself. As a result, if other factors affecting visual results were excluded, the difference in visual outcome for endophthalmitis between postoperative and posttraumatic groups was not significant.

In the analysis according to the cause, removal of the crystalline lens (or IOL) including capsulectomy was performed more frequently in the posttraumatic group than in the postoperative group. In patients with trauma, globe injury, including lens and capsule rupture, was predicted, and limited visualization during vitrectomy was assumed. Therefore, we thought that removal of lens and capsule could provide improved visualization, and this procedure was performed more frequently in patients with trauma. Culture-positive rate was lower in the posttraumatic group than the postoperative group. The culture positivity result depends on the method of sample collection and inoculation, previous medical therapy, and culture medium, and these factors might have contributed to the culture result. The lower rate of positive cultures in the posttraumatic group may be related to the early administration of intravenous antibiotics before the first treatment after globe injury prior to the collection of intraocular samples [29, 30].

Mean time to surgery, initial BCVA, additional surgical treatment, and microorganism species were relevant factors of visual prognosis. First, a number of case series have reported good outcomes following early PPV, and the proportion of patients achieving final visual acuity of 20/40 varied from 23% to 91% [10, 31–33]. Moreover, surveys of ophthalmologists have found that the majority perform early PPV if there is clinical worsening within 48 hours following injection of antibiotics [34]. In our study, 78.3% of patients achieved a final visual acuity better than 20/200 following early PPV within 48 hours. Initial BCVA was related to the progression of endophthalmitis and higher risk of advanced endophthalmitis without a favorable visual outcome as lower initial BCVA. It was already known that the need for an additional procedure was a marker of more severe disease [35]. Eyes that underwent additional surgical treatment showed a tendency to have insufficient effects of initial PPV with SO tamponade. It was presented as advanced endophthalmitis with reaccumulation of inflammatory cells, exudates, and hypopyon. As a result, it was necessary to perform additional surgical treatment but showed unfavorable visual outcomes.

There was a difference in prognosis according to the type of microorganism grown in other results [16, 28]. This study also showed relatively poor visual outcome with *Enterococcus faecalis* and *Pseudomonas aeruginosa* infection. Interestingly, infection with *Staphylococcus* and *Streptococcus* species could generally be expected with favorable visual prognosis and do not need additional treatments to control the infection. Despite the fact that the common antibiotics applied for endophthalmitis had high rate of susceptibility for both *Staphylococcus* and *Streptococcus* infection, the functional outcomes in *Streptococcus* infections were usually worse than *Staphylococcus* infections [36]. But, there was no significant difference of outcomes between the two microorganisms in this study. The poor outcome of *Enterococcus faecalis* and *Pseudomonas aeruginosa* infection is in accordance with other studies: 3 of 8 eyes infected with *Enterococcus faecalis* and 1 of 2 eyes infected with *Pseudomonas aeruginosa* underwent additional surgical treatment. However, the comparison must be evaluated carefully as the number of patients is relatively small and does not allow statistical analysis.

We enrolled in this study only patients who underwent PPV with SO tamponade. Thus, patients treated with intravitreal injection of antibiotics or PPV without SO tamponade were not included. Therefore, enrolled patients tend to have a severe stage of endophthalmitis, and the visual prognosis was thought to be relatively unfavorable compared to the EVS results. Nevertheless, this study has significance in analyzing the long-term effects and prognosis of SO tamponade in patients with exogenous bacterial endophthalmitis.

## 5. Conclusions

In conclusion, this study results suggest a favorable functional outcome in eyes with severe postoperative and posttraumatic endophthalmitis using a SO tamponade with regard to infection control and the need for additional surgery. Therefore, PPV with SO tamponade may be an effective surgical treatment strategy for exogenous bacterial endophthalmitis. We compared postoperative and posttraumatic endophthalmitis, and there was no difference in visual prognosis between subgroups as a long-term result. In addition, mean time to surgery, initial visual acuity, additional surgical treatment, and type of microorganism are significantly related to visual prognosis. Based on these results, it is thought that these factors could be used as a predictor of visual prognosis in patients with endophthalmitis.

## Figures and Tables

**Table 1:** Clinical characteristics of cases with exogenous endophthalmitis.

Characteristics	Value
Sex, *n* (%)	
Male	38 (66.7%)
Female	19 (33.3%)
Age, years	60.7 ± 18.4
Time from sign of endophthalmitis to surgery, days (range)	1.9 ± 2.5 (0–14)
Mean follow-up periods, months (range)	120.8 ± 67.4 (12.6–230.6)
Cause of endophthalmitis, *n* (%)	
Posttraumatic	2 (3.5%)
Globe rupture	6 (10.5%)
Penetrating corneal injury	7 (12.3%)
Intraocular foreign body	
Postoperative	
Cataract surgery	31 (54.3%)
Glaucoma surgery	6 (10.5%)
Vitreoretinal surgery	4 (7.0%)
Intravitreal injection	1 (1.8%)
Lens status, *n* (%)	
Phakia	18 (31.5%)
Pseudophakia	39 (68.5%)
Sclerotomy size, *n* (%)	
20-gauge	21 (36.8%)
23-gauge	36 (63.2%)
Removal of lens and capsule, *n* (%)	31 (54.4%)
Additional surgery after initial vitrectomy, *n* (%)	13 (22.8%)
Intravitreal injection	8 (14.0%)
Anterior chamber irrigation	5 (8.8%)
Prognosis, *n* (%)	
Silicone oil removal surgery	38 (66.7%)
Silicone oil retention eye	17 (29.8%)
Enucleation surgery	2 (3.5%)

Values are presented as the mean ± standard deviations.

**Table 2:** Clinical outcomes between postoperative and posttraumatic groups.

	Postoperative	Posttraumatic	*P* value
Microbial growth			0.019^*∗*^
Positive	26	4	
Negative	16	11	
Removal of lens and capsule			0.003^*∗*^
Yes	18	13	
No	24	2	
Additional surgery after initial vitrectomy			0.760^†^		
Yes	10	3	
No	32	12	
Postoperative status			0.635^‡^
Silicone oil removal surgery	26	12	
Silicone oil retention eye	14	3	
Enucleation/evisceration surgery	1	1	

^*∗*^Data expressed as count and tested by the chi-square test; ^†^data expressed as count and tested by Fisher's exact test; ^‡^data expressed as count and tested by linear-by-linear association.

**Table 3:** Microbiological spectrum.

Bacterial species	Postoperative	Posttraumatic	Total
Gram-positive bacteria			
*Staphylococcus aureus*	1	1	2
*Staphylococcus epidermidis*	6	1	7
*Staphylococcus xylosus*	1	0	1
*Staphylococcus hominis*	1	0	1

Alpha-hemolytic *streptococcus*	6	1	7
*Streptococcus agalactiae*	2	0	2

*Enterococcus faecalis*	7	1	8

Gram-negative bacteria			
*Pseudomonas aeruginosa*	2	0	2

**Table 4:** Factor associated with final visual acuity.

Factor	Final visual acuity		*P* value
	Better than 20/200 (%)	Worse than 20/200 (%)	
Age, years			0.300^*∗*^
Median, range	66, 25–86	60.5, 3–92	
Gender			
Male	14 (36.8%)	24 (63.2%)	0.445^†^
Female	9 (47.4%)	10 (52.6%)	
Time from sign to surgery			0.032^†^
18 (78.3%)	5 (21.7%)		
>48 h	17 (50.0%)	17 (50.0%)	
Vitrectomy instrument			0.344^†^
20-G	12 (35.3%)	22 (64.7%)	
23-G	11 (47.8%)	12 (52.2%)	
Initial BCVA			0.007^‡^
>Hand motion	8 (88.9%)	1 (11.1%)	
≤Hand motion	17 (35.4%)	31 (64.6%)	
Removal of lens and capsule			0.783^†^
Yes	12 (38.7%)	19 (61.3%)	
No	11 (42.3%)	15 (57.7%)	
Additional surgery after initial vitrectomy			0.006^†^
Yes	1 (7.7%)	12 (92.3%)	
No	22 (50.0%)	22 (50.0%)	
Growth of culture			0.629^†^
Positive	13 (43.3%)	17 (56.7%)	
Negative	10 (37.0%)	17 (63.0%)	
Microorganism			0.014^§^
*Staphylococcus* species	7 (63.6%)	4 (36.4%)	
*Streptococcus* species	5 (55.6%)	4 (44.4%)	
*Enterococcus faecalis*	1 (12.5%)	7 (87.5%)	
*Pseudomonas aeruginosa*	0 (0.0%)	2 (100.0%)	

^*∗*^Data expressed as median and range and compared by the Mann–Whitney test; ^†^data expressed as count and percentage and tested by the chi-square test; ^‡^data expressed as count and percentage and tested by Fisher's exact test; ^§^data expressed as count and percentage and tested by linear-by-linear association.

## Data Availability

The datasets used and/or analyzed during the current study are available from the corresponding author upon request.
